# Novel mutations of *PKD* genes in the Czech population with autosomal dominant polycystic kidney disease

**DOI:** 10.1186/1471-2350-15-41

**Published:** 2014-04-03

**Authors:** Lena Obeidova, Veronika Elisakova, Jitka Stekrova, Jana Reiterova, Miroslav Merta, Vladimir Tesar, Frantisek Losan, Milada Kohoutova

**Affiliations:** 1 Institute of Biology and Medical Genetics of the First Faculty of Medicine and General University Hospital, Albertov 4, 128 00 Prague, Czech Republic; 2 Department of Nephrology of the First Faculty of Medicine and General University Hospital, U Nemocnice 2, 128 08 Prague, Czech Republic; 3 Genetika Plzeň, s.r.o., Parková 1254/11a, 326 00 Plzeň-Černice, Czech Republic

**Keywords:** ADPKD, *PKD* gene, Mutational analysis, Mutation, HRM, MLPA, Polycystic kidney disease

## Abstract

**Background:**

Autosomal dominant polycystic kidney disease (ADPKD) is the most common hereditary renal disorder caused by mutation in either one of two genes, *PKD1* and *PKD2*. High structural and sequence complexity of *PKD* genes makes the mutational diagnostics of ADPKD challenging. The present study is the first detailed analysis of both *PKD* genes in a cohort of Czech patients with ADPKD using High Resolution Melting analysis (HRM) and Multiplex Ligation-dependent Probe Amplification (MLPA).

**Methods:**

The mutational analysis of *PKD* genes was performed in a set of 56 unrelated patients. For mutational screening of the *PKD1* gene, the long-range PCR (LR-PCR) strategy followed by nested PCR was used. Resulting PCR fragments were analyzed by HRM; the positive cases were reanalyzed and confirmed by direct sequencing. Negative samples were further examined for sequence changes in the *PKD2* gene by the method of HRM and for large rearrangements of both *PKD1* and *PKD2* genes by MLPA.

**Results:**

Screening of the *PKD1* gene revealed 36 different likely pathogenic germline sequence changes in 37 unrelated families/individuals. Twenty-five of these sequence changes were described for the first time. Moreover, a novel large deletion was found within the *PKD1* gene in one patient. Via the mutational analysis of the *PKD2* gene, two additional likely pathogenic mutations were detected.

**Conclusions:**

Probable pathogenic mutation was detected in 71% of screened patients. Determination of *PKD* mutations and their type and localization within corresponding genes could help to assess clinical prognosis of ADPKD patients and has major benefit for prenatal and/or presymptomatic or preimplantational diagnostics in affected families as well.

## Background

ADPKD is a hereditary renal disorder with the incidence of 1:400 to 1:1000 live births, characterized by focal development and progressive enlargement of cysts in kidneys leading to the decline of renal function. End-stage renal disease (ESRD) occurs during the late phase of the illness and the majority of patients have to start dialysis and/or undergo kidney transplantation. The disease is frequently accompanied by many extra-renal symptoms, such as hypertension, formation of cysts in liver or pancreas, abnormalities of cardiovascular system or development of brain-aneurysms [1].

ADPKD is genetically determined by two genes, *PKD1* (16p13.3) [2] and *PKD2* (4q21-22) [3]. In clinical identified populations, mutations of the *PKD1* gene account for approximately 85% of all ADPKD cases and are responsible for more serious form of the disease (the average age at the onset of ESRD is 58) [4]. Fifteen percent of ADPKD patients with milder clinical presentation (the average age at the onset of ESRD is 79) carry mutation in the *PKD2* gene [5,6]. The hypothetical existence of the third *PKD* locus has not been supported by new reports [7,8].

The *PKD1* gene (46 exons) encodes a large, transmembrane protein called polycystin-1 (4303 amino acids). The *PKD2* gene product (15 exons) is an integral membrane protein polycystin-2 (968 amino acids) that acts as a transient receptor potential (TRP) ion channel involved in the regulation of intracellular Ca^2+^ concentration. Both proteins interact with each other in kidney primary cilia forming complex that hypothetically functions as a flow-dependent mechanosensor with a crucial role in the adhesion, proliferation and differentiation of tubular epithelial cells [9,10].

Both genes are highly polymorphic and sequence changes are spread over their whole length [11]. The majority of mutations are unique to a single family, recurrent mutations account only for 30% of the total [12]. Currently, 978 sequence variants of the *PKD1* gene and 193 sequence variants of the *PKD2* gene have been published in the Human Gene Mutation Database (HGMD) (http://www.hgmd.cf.ac.uk). The specialized Autosomal Dominant Polycystic Kidney Disease Mutation Database (PKDB) presents even 1,923 different sequence variants of *PKD1* and 241 sequence variants of the *PKD2* gene (http://pkdb.mayo.edu; [13]).

The high variability of sequence changes is not the only fact that makes the analysis of *PKD* genes difficult. First 33 exons of the *PKD1* gene are duplicated six times with 97% sequential identity. These pseudo genes are located in the close surroundings of the original gene and significantly complicate the mutational screening of the *PKD1* gene [14].

The clinical course of ADPKD is highly variable not only among families with different mutations but within families with a defined mutation as well. The large phenotypic variability of ADPKD within affected families highlights a role for the genetic background in disease presentation. Hypomorphic or incompletely penetrant *PKD1* or *PKD2* alleles have been described, causing mild cystic disease [15,16]. Coinheritance of hypomorphic allele in combination with an inactivating *PKD* allele can be associated with early-onset disease. About 2% of ADPKD patients show such an early and severe phenotype (renal enlargement *in utero* or in infancy) that can be clinically indistinguishable from autosomal recessive polycystic kidney disease (ARPKD). These severe cases have been explained by the presence of the expected familial germ-line mutation and, in addition, other supplementary mutations in *PKD* genes (including *PKD1*, *PKD2*, *PKHD1* and also *HNF-1β* gene), which act as modifying genes and likely aggravate the final phenotype [17]. Moreover, mosaicism can also modulate the course of ADPKD in some families [16,18,19].

Diagnosis of ADPKD is usually based on an age-specific phenotype of affected kidneys (number and size of renal cysts on the ultrasound, computed tomography or magnetic resonance imaging), accompanying factors (for example hypertension) and positive family history [19-21]. Molecular diagnostics (traditional linkage analysis or direct mutational screening) is a useful tool to determine and confirm the definite diagnosis, especially for evaluation of potential living related kidney donors, for individuals with negative family history and for patients with early onset of ADPKD [22]. Molecular analysis of *PKD* genes is quite complicated and time-consuming, therefore new methods are being developed to simplify the whole process [23-25].

In the present study, the mutational analysis of both *PKD* genes in a cohort of 56 Czech patients with ADPKD was performed using HRM and MLPA techniques.

## Methods

### Patients

The mutational screening of *PKD* genes was performed in a set of 56 unrelated Czech patients with ADPKD diagnosed by renal ultrasound in accordance with previously described criteria [26]. The study was approved by the Ethics Committee of General University Hospital in Prague on May 23, 2006. All patients provided written informed consent. According to the linkage analysis, the disease was linked to the *PKD1* gene in 20 individuals. In 26 patients a relation of the disease to the *PKD1* gene was not proved and 10 patients had an isolated occurrence of the disease within a family.

### Linkage analysis

Linkage analysis was carried out using a panel of CA-repeat flanking markers. The combination and number of markers used for analysis were selected to achieve at least two informative markers, one on the 5′end and the second on the 3′end of *PKD* gene to rule out possible recombination. The markers used for *PKD1* analysis were: D16S521, D16S3024, KG8, CW4, CW3, CW2, and for *PKD2* analysis: D4S231, D4S1534, JSTG3, JV108, AICA1, D4S1563 and D4S414.

### Mutational screening

The genomic DNA was isolated from the peripheral blood lymphocytes using QIAGEN spin columns on a QIAcube (QIAGEN GmbH).

At first, the screening of the *PKD1* gene was carried out in the selected set of 56 patients. For mutational analysis of the *PKD1* gene, the technique of a long-range PCR (LR-PCR) followed by nested PCR was used [27]. Nested PCR amplicons were produced and analyzed by the method of High Resolution Melting (HRM); positive clones were reanalyzed and confirmed by sequencing in both directions. The protocol of the whole strategy (including primers and PCR reaction conditions) was previously described in detail by Reiterova *et al*. [16].

The DNA of patients where no definite pathogenic mutation was found within the *PKD1* gene (29 samples) were subsequently analyzed via mutational screening of the *PKD2* gene by HRM and via MLPA for large rearrangements of both *PKD1* and *PKD2* genes. This subset of 29 individuals included patients without any sequence changes found by mutational analysis of the *PKD1* gene as well as patients with unpublished putative missense mutations, putative splice-site mutations and in-frame deletions/insertions detected in the *PKD1* gene.

**Figure 1:**
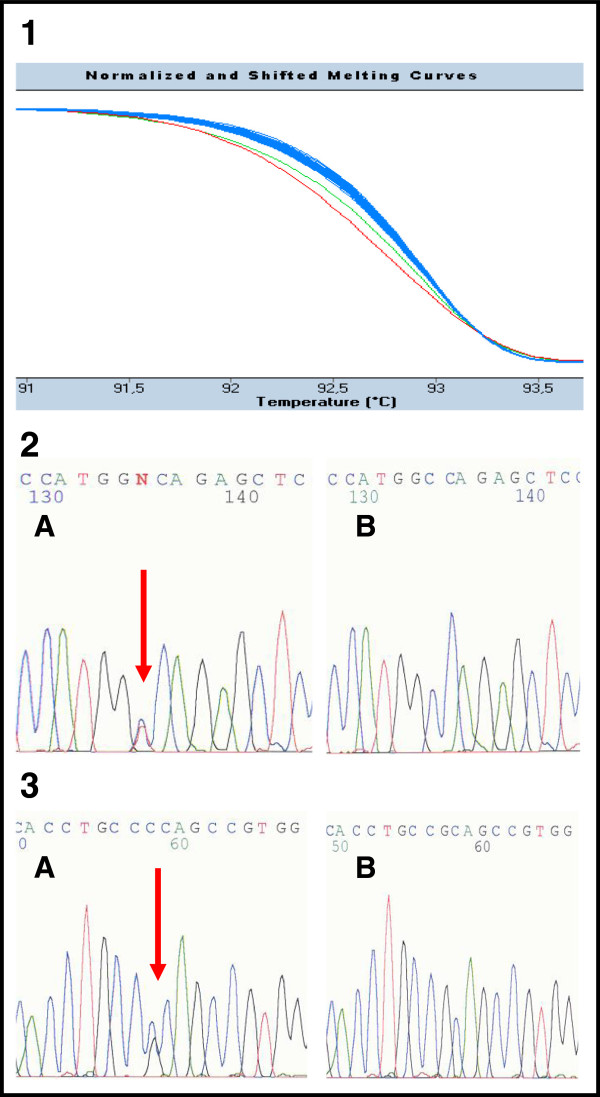
**The example of HRM analysis in patients 385 and 419 (exon 39). 1** Detection of mutations by HRM. The green curve in the diagram corresponds to the PCR fragment carrying the nonsense mutation c.11172G > A (p.Trp3724X) in patient 385. The red curve refers to the PCR fragment carrying the likely pathogenic substitution c.11248C > G (p.Arg3750Gly) in patient 419. The rest of fragments with blue curves are wild type samples (patients without sequence changes). **2, 3** Confirmation of mutations by direct sequencing in patient 385 (number 2) and patient 419 (number 3). Both sequences are in reverse direction. The letter A corresponds to the mutated sequence; B is the wild type.

The coding region and intron-exon boundaries of the *PKD2* gene were screened via 17 PCR fragments. The sequences of PCR primers (some primers were designed on the base of Tan *et al.*) and the conditions of PCR reactions are available upon request [28]. PCR fragments were produced and analyzed by HRM using the LightCycler® 480 (Roche Applied Science). The reaction mixture (total volume of 10 μl) contained 1 μl of diluted template DNA, 1 × LightCycler® 480 High Resolution Melting Master Mix (Roche Applied Science) and 5 pmol of primers. The LightCycler® 480 Gene Scanning Software (Roche Applied Science) analyses the melting profile of samples to identify changes in the shape of melting curves, which indicate both homozygous and heterozygous allelic variants in a sample (Figure 1) [29]. All PCR fragments showing aberrant melting curves by HRM were sequenced. The sequencing reaction was performed according to the manufacturer’s instructions using the BigDye® Terminator v1.1 Cycle Sequencing Kits (Applied Biosystems) and ran on an ABI PRISM® 310 Genetic Analyser (Applied Biosystems).

MLPA analysis (MRC-Holland) was carried out with SALSA MLPA KIT P351-B1/P352-B1 PKD1-PKD2, which together covers 37 from 46 exons of the *PKD1* gene, its upstream regulatory sequences, entire *PKD2* gene and three exons of the *TSC2* gene adjacent to the *PKD1* gene [30]. The MLPA reaction was performed with 50–100 ng of genomic DNA according to the manufacturer’s instructions. The result of MLPA was carried out by fragmentation analysis with the use of ABI PRISM® 310 Genetic Analyser (Applied Biosystems). To obtain the final results, the generated raw data were normalized according to the manufacturer’s instructions. At first intra-sample normalization was performed by dividing the peak area of each probe’s amplification product by the total area of only the reference probes in the probemix. Subsequently, the normalization between individual samples was done by dividing the intra-normalized probe ratio of each sample by the average intra-normalized probe ratio of reference samples (negative controls). In-house developed method was used for the entry of all these calculations.

To prove the segregation of the potential mutation in *PKD* genes with the disease in the family, the DNAs of all available members of the respective family (including healthy individuals) were tested as well.

### Data analysis and sequence changes classification

All sequenced samples were compared to the following NCBI reference sequences: [GeneBank: NM_001009944.2] for the *PKD1* gene and [GeneBank: NM_000297.3] for the *PKD2* gene. The sequence changes found within this study were checked with the list of *PKD* sequence variants currently published in the Human Gene Mutation Database (HGMD) (http://www.hgmd.cf.ac.uk) and the Autosomal Dominant Polycystic Kidney Disease Mutation Database (PKDB) (http://pkdb.mayo.edu). In case of novel mutations, their pathogenic potential was assessed. Nonsense or frameshift variants leading to a premature STOP codon were considered as definitely pathogenic.

The pathogenic potential of missense variants was evaluated computationally by examining interspecies sequence variations using PolyPhen2 (http://genetics.bwh.harvard.edu/pph2/; [4,31,32]) and Sorting Intolerant from Tolerant (SIFT) (http://sift.bii.a-star.edu.sg/; [4,32,33]) software. The sequence variant was considered as likely pathogenic, if both scores assessed its pathogenity. When only one test predicted the pathogenic potential, the variation was reported as indeterminate. The pathogenic relevance of putative splice site mutations was evaluated in the same way using NetGene2 (http://www.cbs.dtu.dk/services/NetGene2/; [34]) and Human Splicing Finder (HSF) (http://www.umd.be/HSF/; [35]) software. Further criteria for classification of missense or splice site variant as likely pathogenic mutation were its segregation within the affected family and absence of additional, more probable mutation in a concrete patient (family).

Possible influence of sex and type of mutations on the age of renal failure was evaluated. For this purpose, Shapiro-Wilk normality test for each group was performed and subsequently, according to results of normality test, unpaired two-sample t-test was used to compare the groups. All computations were carried out using in-house developed scripts in R language.

## Results

We performed mutational analysis in *PKD* genes in a set of 56 unrelated Czech patients with ADPKD diagnosed by renal ultrasound. Twenty selected individuals came from families where ADPKD was linked unequivocally to the *PKD1* gene according to the linkage analysis. Other 26 cases were the families with more serious manifestation of the disease (ESRD before 60 years of age). In this group of patients, a direct relation of the disease to the *PKD1* gene has not been proven by the method of linkage analysis. At last, 10 unrelated individuals with an isolated occurrence of the disease within their families were examined (an assumption of *de novo* mutation).

Mutational screening of the *PKD1* gene revealed 38 (36 different) likely pathogenic sequence changes in 37 from the total set of 56 patients (Table 1). In one individual, two different likely pathogenic mutations were found.

**Table 1:** **Likely pathogenic sequence changes of the *PKD1 *** **gene identified by HRM and direct sequencing in a set of patients from the Czech population**

**Family**	**Exon/intron**	**Nucleotide change**	**Amino acid change/predicted effect**	**Predicted location within *PKD1 *** **domains**	**Reference**
**394 **^***ISOL.***^	**4**	**c.450_459del10**	**p.Glu151ArgfsX135**	LRRNT	**Novel**
**338 **^***PKD1***^	**7**	**c.1564 T > C**	**p.Cys522Arg**	C-type lectin domain	**Novel**
39 ^*INDET.*^	15	c.3312_3313dupC	p.Val1105ArgfsX4	PKD repeats	[16]
**429 **^***PKD1***^	**15**	**c.4551_4581del31**	**p.Tyr1517X**	PKD repeats	**Novel**
**236 **^***PKD1***^	**15**	**c.5650G > T**	**p.Glu1884X**	PKD repeats	**Novel**
478 ^*INDET.*^	15	c.5824_5825dupC	p.Arg1942ProfsX47	PKD repeats	[36]
301 ^*PKD1*^	15	c.5995G > A	p.Gly1999Ser	PKD repeats	[12]
496 ^*ISOL.*^					
**315 **^***PKD1***^	**15**	**c.6130A > G**	**p.Asn2044Asp**	PKD repeats	**Novel**
**249 **^***INDET.***^	**15**	**c.6586C > T**	**p.Gln2196X**	REJ domain	**Novel**
**427 **^***ISOL.***^	**15**	**c.6895delG**	**p.Ala2299ProfsX14**	REJ domain	**Novel**
**237 **^***PKD1***^	**16**	**c.6960_6961insG**	**p.Ser2321GlufsX98**	REJ domain	**Novel**
253 ^*PKD1*^	16	c.7000_7001dupGCTGGCG	p.Val2334GlyfsX87	REJ domain	[37]
**246 **^***PKD1***^	**17**	**c.7144_7156del13insTAC**	**p.Ser2382TyrfsX234**	REJ domain	**Novel**
**112 **^***INDET.***^	**18**	**c.7484G > A**	**p.Cys2495Tyr**	REJ domain	**Novel**
**403 **^***PKD1***^	**20**	**c.7758_7761del4**	**p.Trp2587CysfsX31**	REJ domain	**Novel**
**357 **^***INDET.***^	**20**	**c.7856 T > G**	**p.Leu2619Arg**	REJ domain	**Novel**
438 ^*ISOL.*^	23	c.8309A > T	p.Asn2770Ile	REJ domain	[36]
336 ^*INDET.*^	23	c.8311G > A	p.Glu2771Lys	REJ domain	[37]
389 ^*PKD1*^					
275 ^*ISOL.*^	23	c.8428G > T	p.Glu2810X	REJ domain	[38]
		c.8614delA	p.Ile2872SerfsX3		[39]
**482 **^***INDET.***^	**IVS23**	**c.8792-2A > T**	**Probable splice defect**	N/A	**Novel**
**479 **^***INDET.***^	**27**	**c.9416_9417dupACGTGGG**	**p.Ile3140ArgfsX40**	PLAT domain	**Novel**
**430 **^***PKD1***^	**28**	**c.9584G > A**	**p.Trp3195X**	PLAT domain	**Novel**
**391 **^***PKD1***^	**29**	**c.9904G > C9906_9907delTG**	**p.Val3302LeufsX86**	TM domain	**Novel**
**395 **^***ISOL.***^	**33**	**c.10321C > T**	**p.Gln3441X**	Not defined	**Novel**
**413 **^***INDET.***^	**IVS36**	**c.10821 + 4_10821 + 6delAGG**	**Probable splice defect**	N/A	**Novel**
330 ^*INDET.*^	37	c.10951G > A	p.Gly3651Ser	Not defined	[40]
**297 **^***PKD1***^	**37**	**c.10980_10982delAGC**	**p.Glu3660_Ala3661delinsAsp**	Not defined	**Novel**
**385 **^***INDET.***^	**39**	**c.11172G > A**	**p.Trp3724X**	PKD channel domain	**Novel**
**419 **^***INDET.***^	**39**	**c.11248C > G**	**p.Arg3750Gly**	PKD channel domain	**Novel**
379 ^*INDET.*^	41	c.11482_11484delGAG	p.Glu3828del	PKD channel domain	[40]
**420 **^***PKD1***^	**43**	**c.11884 C > T**	**p.Gln3962X**	PKD channel domain	**Novel**
**161 **^***INDET.***^	**43**	**c.11993_11994dup9**	**p.Leu3998_Leu3999insPheLeuLeu**	PKD channel domain	**Novel**
**425 **^***INDET.***^	**IVS** **43-44**	**c.12004-15_12015del27**	**Probable splice defect**	N/A	**Novel**
215 ^*PKD1*^	44	c.12061 C > T	p.Arg4021X	PKD channel domain	[41]
**454 **^***INDET.***^	**45**	**c.12439_12441delAAG**	**p.Lys4147del**	Not defined	**Novel**

cDNA numbering is based on the reference database: Autosomal Dominant Polycystic Kidney Disease Mutation Database (PKDB) (http://pkdb.mayo.edu). Novel probable mutations are in **boldface** type. *ISOL.* patients with isolated occurrence of ADPKD in a family; *PKD1* patients with proved linkage of ADPKD to the *PKD1* gene; *INDET.* indeterminate patients (the linkage of ADPKD to *PKD1* gene has not been proved); IVS – the intronic sequence; Novel mutation was not described in the Autosomal Dominant Polycystic Kidney Disease Mutation Database (PKDB) (http://pkdb.mayo.edu) and/or in the Human Gene Mutation Database (HGMD) (http://www.hgmd.cf.ac.uk). The potential location of mutations has been established on the basis of theoretical model of polycystin-1 by UniProtKB/Swiss-Prot database [P98161]. LRRNT – leucine rich repeat N-terminal domain; REJ – receptor for Egg Jelly; PLAT – Polycystin-1, Lipoxygenase, Alpha-Toxin domain; TM – transmembrane domain. As not defined are called sequences within *PKD1* protein with unknown domain structure.

In total, eleven frameshift mutations (29%), nine nonsense mutations (24%), eleven missense mutations (29%), four small in-frame deletions/insertions (10%) and three putative splice-site mutations (8%) were detected in our set of patients. Via MLPA, one additional new large deletion was found within the *PKD1* gene.

Fifty-seven percent (21) of likely pathogenic mutations found matched the definitions of being definitely pathogenic. Twenty-six of the mutations found in this study were novel. Moreover, three additional indeterminate sequence changes (two missense and one splice-site) were described (Table 2).

**Table 2:** **Indeterminate sequence changes of *PKD *** **genes identified by HRM and direct sequencing in a set of patients from the Czech population**

**Family**	**Gene/exon/intron**	**Nucleotide change**	**Amino acid change/predicted effect**	**Predicted location within *PKD *** **domains**	**Reference**
308 ^*INDET.*^	*PKD1*/15	c.3602C > T	p.Ala1201Val	PKD repeats	Novel
412 ^*INDET.*^	*PKD1*/29	c.9718G > A	p.Ala3240Thr	Not defined	Novel
409 ^*INDET.*^	*PKD1*/IVS42	c.11712 + 8C > A	Probable splice defect	N/A	Novel
466 ^*INDET.*^	*PKD2*/IVS 9	c.2019 + 9A > C	Probable splice defect	N/A	Novel

cDNA numbering is based on the reference database: Autosomal Dominant Polycystic Kidney Disease Mutation Database (PKDB) (http://pkdb.mayo.edu). *INDET.* indeterminate patients (the linkage of ADPKD to *PKD1* gene has not been proved); IVS the intronic sequence; Current paper mutation was not described in the Autosomal Dominant Polycystic Kidney Disease Mutation Database (PKDB) (http://pkdb.mayo.edu) and/or in the Human Gene Mutation Database (HGMD) (http://www.hgmd.cf.ac.uk). The potential location of mutations has been established on the basis of theoretical models of polycystins by UniProtKB/Swiss-Prot database (*PKD1*: P98161, *PKD2*:Q13563). As not defined are called sequences within *PKD* proteins with unknown domain structure.

A great deal of sequence variants of the *PKD1* gene detected in our study were changes which do not alter the amino acid composition or have been already published as polymorphisms (or sequence changes with neutral effect on the function of polycystin 1). Complementary mutational analysis of the *PKD2* gene revealed two already described likely pathogenic mutations and one indeterminate splice-site variant. All three sequence changes were found within patients without proven linkage of ADPKD to the *PKD1* gene (Table 2 and 3). Screening of the *PKD2* gene thus increased the final detection level of likely pathogenic mutations in this study to 71% (40 patients with a putative mutation from the set of 56 individuals in total).

**Table 3:** **Likely pathogenic sequence changes of the *PKD2 *** **gene identified by HRM and direct sequencing in a set of patients from the Czech population**

**Family**	**Exon/intron**	**Nucleotide change**	**Amino acid change**	**Predicted location within *PKD2 *** **domains**	**Reference**
421 ^*INDET.*^	6	c.1345_1346insGCAACAG	Gly450AsnfsX22	Polycystin cation channel domain	[42]
467 ^*INDET.*^	IVS 11	c.2240 + 1G > T	Splice	N/A	[43]

cDNA numbering is based on the reference database: Autosomal Dominant Polycystic Kidney Disease Mutation Database (PKDB) (http://pkdb.mayo.edu). *INDET.* indeterminate patients (the linkage of ADPKD to *PKD1* gene has not been proved); IVS the intronic sequence. The potential location of mutations has been established on the basis of theoretical model of polycystin-2 by UniProtKB/Swiss-Prot database [Q13563].

Regarding the distribution of revealed mutations among the patients with distinctive phenotype and a family history, 16 likely pathogenic mutations (42%) were found in a cohort with more serious manifestation of ADPKD (ESRD reached before the age of 60 years). Fifteen putative mutations (approximately 40%) were detected among families with clear linkage to the *PKD1* gene. The rest (18% of likely pathogenic mutations) referred to the set of patients without positive family history (isolated occurrence in a family).

From all 56 patients and their analyzed affected relatives, 20 males and 17 females have already reached ESRD (Table 4). Computation of dependency of sex and type of mutation (missense and small in-frame indels vs. truncating mutations) on the age of ESRD was performed in a group of 37 individuals. According to the Shapiro-Wilk test of normality, all tested groups had normal distribution (p > 0.1). Two-sample t-test did not reject null hypothesis in either sex (t = −0.9, p = 0.4), or type of mutation (t = 0.8, p = 0.4) dependency on age of renal failure.

**Table 4:** **Ages of end stage renal disease in families with likely pathogenic mutation found in the *PKD1 *** **gene**

**Family**	**Nucleotide change**	**Amino acid change/predicted effect**	**Ages of end stage renal disease in the family in years, M-male, F-female**
429	c.4551_4581del31	p.Tyr1517X	M 39, M 26
236	c.5650G > T	p.Glu1884X	M 52
478	c.5824_5825dupC	p.Arg1942ProfsX47	M 40
301	c.5995G > A	p.Gly1999Ser	M 54
237	c.6960_6961insG	p.Ser2321GlufsX98	M 48, F 45
253	c.7000_7001dupGCTGGCG	p.Val2334GlyfsX87	M 61, M 60, M 36
403	c.7758_7761del4	p.Trp2587CysfsX31	F 60
357	c.7856 T > G	p.Leu2619Arg	M 41, F 57
482	c.8792-2A > T	Probable splice defect	M 43
479	c.9416_9417dupACGTGGG	p.Ile3140ArgfsX40	F 79, M 55, M 51
430	c.9584G > A	p.Trp3195X	M 57
391	c.9904G > C9906_9907delTG	p.Val3302LeufsX86	M 61
413	c.10821 + 4_10821 + 6delAGG	Probable splice defect	F43, F 49
330	c.10951G > A	p.Gly3651Ser	F73, F71, M 73
297	c.10980_10982delAGC	p.Glu3660_Ala3661delinsAsp	M 41, F 44, F 35
385	c.11172G > A	p.Trp3724X	F 49
419	c.11248C > G	p.Arg3750Gly	F 44
420	c.11884 C > T	p.Gln3962X	F 45, F 46
425	c.12004-15_12015del27	Probable splice defect	F 65, F 51
215	c.12061 C > T	p.Arg4021X	F 58, M 50, M 50
454	c.12439_12441delAAG	p.Lys4147del	M 67

### Nonsense and frameshift mutations

One patient (275) from our study was found to have two truncating pathogenic mutations (one nonsense mutation and one frameshift mutation), both already published and both located within the exon 23 probably in cis regarding his mild phenotype of ADPKD without progression. Similar cases have been already described in the literature, but their existence is rare [11]. The patients 394, 395 and 427 are the representatives of the group with isolated occurrence of ADPKD within their families.

### Missense mutations

Via our mutational analysis of both *PKD* genes, 9 likely pathogenic missense mutations have been identified; all within the *PKD1* gene. Five mutations have been determined as novel and two already published substitutions (p.Gly1999Ser and p.Glu2771Lys) were recurrent (both present in two different individuals). Eight substitutions segregated with the disease in affected families (Table 5) and were confirmed as pathogenic by both predictor programs (PolyPhen2 and SIFT), one substitution occurred *de novo*.

**Table 5:** **Segregation of likely pathogenic missense and small in-frame indel mutations of *PKD1 *** **with polycystic kidney disease in affected families**

**Amino acid change**	**Nucleotide change**	**Family**	**Number of affected/healthy family members**
p.Cys522Arg	c.1564 T > C	338	5/2
p.Gly1999Ser	c.5995G > A	301	3/2
p.Asn2044Asp	c.6130A > G	315	6/4
p.Cys2495Tyr	c.7484G > A	112	3/3
p.Leu2619Arg	c.7856 T > G	357	2/3
p.Glu2771Lys	c.8311G > A	336	2/1
		389	6/5
p.Gly3651Ser	c.10951G > A	330	2/3
p.Arg3750Gly	c.11248C > G	419	2/2
p.Glu3660_Ala3661delinsAsp	c.10980_10982delAGC	297	2/4
p.Leu3998_Leu3999insPheLeuLeu	c.11993_11994dup9	161	3/3
p.Lys4147del	c.12439_12441delAAG	454	3/2

### Splice-site mutations

The probable splice-site mutations c.8792-2A > T (patient 482) and c.12004-15_12015del27 (patient 425) cover the consensus sequence of conserved splice-site and both predictor programs suggested their pathogenity. Although the probable splice-site mutation c.10821 + 4_10821 + 6del AGG (patient 413) is also situated within the conserved splice-site, its pathogenic impact is milder. According to NetGene2 and HSF, this mutation does not abolish the splice-site completely but decreases its efficiency significantly. Therefore this mutation was classified as pathogenic as well.

### Small in-frame deletions/insertions

Within our set of 56 Czech individuals (families), four small in-frame deletions/insertions were detected; three of them were described as novel. The common molecular mechanisms of origin of such genomic events are non-homologous end joining (NHEJ) or microhomology-mediated replication-dependent recombination (MMRDR) [44]. All three novel small in-frame deletions/insertions characterized in the present study show high evolutionary conservation of involved amino acid residues; all three are the only probably pathogenic mutations identified in the patient and segregate with the disease in affected families (Table 5). Their pathogenic potential is therefore highly probable.

### Large rearrangements

By the method of MLPA, one novel large deletion of the *PKD1* gene was revealed in patient 105 with proven linkage of ADPKD to the *PKD1* gene. The deletion was spread from the exon 3 to the first half of exon 15. The exact beginning of the deletion is arguable, because there is no probe for exon 2 in the MLPA kit used within our study. The deletion was identified in all available affected members of the family but was not characterized at the nucleotide level due to the sequence complexity of the *PKD1* locus.

### Indeterminate sequence changes

Four sequence changes found within our set of patients were categorized as indeterminate (Table 2). The possible splice-site mutations *PKD1*/c.11712 + 8C > A and *PKD2*/c.2019 + 9A > C segregate with the disease in affected families and are the only possible mutations found in patients 409 and 466, respectively. However, their location is out of the conventional range of the consensus splice-site sequence and both predictor programs (NetGene2 and HSF) evaluated them as non-pathogenic. Despite its segregation within affected family, also the substitution *PKD1*/p.Ala1201Val (patient 308) was determined as benign by both software programs used (PolyPhen2 and SIFT). Although the substitution *PKD1*/p.Ala3240Thr (patient 412) was predicted as possibly damaging by PolyPhen2, SIFT defined it as benign as well.

## Discussion

The highest portion of mutations found within our study (54%) was predicted to truncate the protein. These truncating changes include frameshift mutations, nonsense mutations and one large deletion. Our findings seem to be in concordance with the recent publication by Cornec-Le Gall *et al.*, where approximately two thirds of *PKD1* mutation-positive pedigrees carried truncating mutations [6]. In addition, the most frequent definitely pathogenic mutation type of the *PKD1* gene in our study is a frameshift mutation (29%), which is consistent with detailed current reports [6,12,36].

### Characterization of mutations

In the present study, only 5.3% of mutations were recurrent (2 different already published mutations, both found in two different unrelated families). Both these mutations occur within CpG dinucleotides (G > A transitions), which are mutational hotspots typical for recurrent sequence changes [45]. The substitution p.Glu2771Lys is even published for the sixth time (always from a different location). This fact strongly supports its recurrent origin instead of the existence of a common ancestor.

Such a low percentage of recurrent mutations is in contrast with studies by other groups. Up to 30% of recurrent mutations were mentioned regarding *PKD* genes in the study by Rossetti *et al.*[12]. Also, the latest comprehensive mutational analysis of *PKD* genes in 700 unrelated patients revealed 20.8% of recurrent mutations [36]. The difference can be caused by a smaller number of patients analyzed within our study (56) compared to extensive studies by Rossetti *et al. *(202) and Audrézet *et al.* (700).

According to the recent data from literature, ADPKD without family history is present in about 10% of adult patients [46]. The clinical characteristics of patients with and without family history are very similar but only about a half of patients without positive family history show extrarenal abnormalities. In our case, 18% of found likely pathogenic mutations fall into the group of patients with isolated occurrence of ADPKD (the so called *de novo* mutations).

Large DNA rearrangements account for a maximum of 4% of all *PKD1* mutations and even fewer of all *PKD2* mutations [18]. Finding of only one large deletion of the *PKD1* gene within our set of patients (approximately 2.5%) is thus in accordance with the data from literature.

### Location of mutations

Sequence changes of all types were spread throughout the *PKD1* gene (Table 1). The only evidence of a cluster of mutations (especially truncations) was at the end of exon 15. This region corresponds to the junction of the PKD repeats and the REJ domain of the resulted protein. The reference about this sort of clustering has been already mentioned in literature by Rossetti *et al.*[12]. No determination of mutational distribution in the *PKD2* gene can be done as only one exonic mutation was found. Both *PKD* genes are characterized by a high level of allelic heterogeneity (a great number of polymorphisms), relatively high GC content and no mutation hotspots. The mutations are usually unique, highly variable and spread throughout the entire gene which fully corresponds with our observations [13]. However, the latest comprehensive study of *PKD* genes suggests the existence of theoretical hotspots for the three major types of mutations within the *PKD1* gene [36]. Our results could not confirm this hypothesis because of the limited extent of the study that did not allow us such a specific computational analysis.

### Genotype/phenotype correlation

Generally, the ADPKD phenotype is very variable and the correlation between genotype and phenotype is usually not apparent, even within one family. One of the explanations could be a different genetic background and the effect of heritable modifying factors.

The influence of gender, mutation type and mutation location on phenotype of ADPKD is limited and not completely clear. A slight correlation between gender and median age at ESRD onset has been described in literature (56.1 years for men versus 59.5 years for women) [6]. Although the influence of gender has been demonstrated in case of the *PKD2* gene too, the most recent report has not proved this dependency [4,6]. Our data also suggested a slightly higher mean age of ESRD in ADPKD females than in males (53.8 ± 12.3 years versus 50.3 ± 11.4 years). The ages of ESRD in Czech patients compared with those described by Cornec-Le Gall *et al.* were slightly lower, but in concordance with observations made by Torra *et al*. (49.6 years for men versus 53.1 years for women) [47].

Another dependency examined within our study was the effect of distinctive type of mutation on the phenotype of ADPKD. We observed that the mean age of ESRD is slightly higher in case of Czech ADPKD patients with missense mutations and in-frame amino acids deletions (54.6 ± 14.4 years) in comparison with patients carrying protein truncating mutations (50.9 ± 11.1 years). This observation corresponds with a strong correlation between the mutation type and median age at ESRD onset that has been recently presented in literature (55.6 years in case of truncating mutations versus 67.9 years in case of mutations with no truncating effect) [6]. For further examination of this dependency a bigger data set would be necessary as only information from 11 patients with missense mutations and in-frame amino acids deletions was available.

Two *PKD1* mutations were confirmed in a 24-year-old patient with normal renal function (patient 275 with two mutations within exon 23). The ultrasound of his parents was normal, without cysts. It seems probable that both mutations were de novo however DNA samples from parents were not available. Moreover, a family with large *PKD1* deletion was described. The age of ESRD in two males (40 years and 53 years) was known in this family. Intrafamiliar variability has been repeatedly described in PKD families, and was noted in this family, as well. The clinical course in the male with ESRD at the age of 40 years was influenced by arterial hypertension. There were three more affected members in this family (35–50 years) with normal renal function or only mild renal insufficiency. The large *PKD1* deletion does not lead to severe clinical course.

The more severe manifestation of the disease in patients with mutations in the 5′ region of the *PKD1* gene has been described in comparison with patients carrying mutations in the 3′ region [11]. Nevertheless, a recent study with 741 patients has not shown the significant difference between the 5′ and 3′ end regions [6]. In the case of the *PKD2* gene, no clear relationship between mutation location and ADPKD severity has been found, either [48]. Within our study, no effect of location of *PKD1* mutations on the mean age of ESRD of Czech ADPKD patients has been observed.

### Detection techniques

Mutation analysis of highly polymorphic *PKD* genes is challenging because of the length of genes (especially *PKD1* gene), multi-exon structures, high GC content, marked allelic heterogeneity and existence of highly homologous pseudogenes of the *PKD1* gene. On average, as much as 10.1 different sequence changes per patient are reported in case of the *PKD1* gene [12,14]. Few complete screens of both genes have been already published with the use of different molecular techniques, but all described methods have still a lot of limitations, including time-demand, high price or insufficient sensitivity. The detection level of the present study is 71% (40 patients/families with likely pathogenic mutations in *PKD* genes from 56 patients in total) which is in accordance with the 75% efficiency reported by Bataille *et al.*, where the same method of high resolution melting (HRM) was used [23]. The cases with no probable mutation found may correspond to allele dropout, different category of sequence changes that have not been analyzed within this study (such as deep intronic variants or missed mutations in promoter regions) or can be caused by incorrect original diagnosis. Hypomorphic variants or polygenic inheritance may also play an important role [15,17]. By denaturing high-performance liquid chromatography (DHPLC), approximately 64% efficiency for definitely pathogenic mutations has been achieved [11]. A current publication concerning DHPLC presented the total detection rate of 52.3% [39]. The combination of DHPLC with direct sequencing has increased the level of detection up to 89% [12]. Similarly, 86% efficiency has been reported with Transgenomic’s SURVEYOR Nuclease and WAVE Nucleic Acid High Sensitivity Fragment Analysis System [28]. Using direct sequencing alone, different results have been achieved ranging from 64.5% of mutations detected by Hoefele *et al.*, 78% of probable pathogenic mutations detected by Garcia-Gonzales *et al.* to the highest efficiency of 93.8% reported by Cornec-Le Gall *et al.*[6,36,49,50]. In the most recent studies, the new high-throughput next generation sequencing (NGS) techniques have been tested for mutational analysis of *PKD* genes with the maximal detection rate of 63% and 69.4% [24,25]. The NGS deep sequencing helps with identification of missed or atypical sequence variants, which are hardly detectable through the conventional Sanger sequencing (especially allele dropout, gene conversion or deep intronic variants). Promising is also the new LR-PCR Sequencing Method developed by Tan *et al.* with very high sensitivity, improved intronic coverage and marked saving of time and costs [51].

In comparison with other detection techniques, HRM analysis has good and comparable efficacy. Ninety-six or even 394 samples could be analyzed at once through the simple strategy of PCR followed automatically by HRM analysis. The most important benefits of this method are thus the savings of time and costs and general convenience of the working strategy, decreasing the probability of mistakes within the whole process.

## Conclusion

The methods for mutational identification are constantly improving and enable us to screen even such a complex gene as *PKD1*. The HRM and MLPA are relatively fast and convenient methods that together reached the detection level of 71%. The question is how to interpret the data from genetic analysis. The differences in severity of the disease within affected relatives suggest that the phenotype of ADPKD is influenced not only by the type or location of the mutation but also by genetic background and environmental factors. The assessment of patient’s prognosis on the basis of molecular diagnostics is therefore still impossible, but allows us a screening of presymptomatic relatives and kidney donors. Full characterization of molecular etiology of ADPKD should facilitate possible therapeutic intervention.

## Competing interests

The authors declare that they have no competing interests.

## Authors’ contributions

VE participated to carry out the molecular genetic studies of *PKD* genes and drafted the manuscript. JS participated in the conception and design of the study and carrying out of molecular genetic studies. LO carried out the mutational screening of *PKD2* gene and revised final manuscript. JR participated in the conception and design of the study and collected the clinical data. MM and FL participated in recruitment of patients and collecting of clinical data. VT and MK conceived the study. All authors read and approved the final manuscript.

## Pre-publication history

The pre-publication history for this paper can be accessed here:

http://www.biomedcentral.com/1471-2350/15/41/prepub
